# Effect of Combination Exercise Therapy on Walking Distance, Postural Balance, Fatigue and Quality of Life in Multiple Sclerosis Patients: A Clinical Trial Study

**DOI:** 10.5812/ircmj.17173

**Published:** 2014-06-05

**Authors:** Bahram Sangelaji, Seyed Massood Nabavi, Fatemeh Estebsari, Mohammad Reza Banshi, Hamideh Rashidian, Ensiyeh Jamshidi, Maryam Dastoorpour

**Affiliations:** 1 Hayat-e-no Physiotherapy Clinic, Iran’s Multiple Sclerosis Society, Tehran, IR Iran; 2 Neurology Department, Mostafa Khomeini Hospital, Shahed University, Tehran, IR Iran; 3 Department of Health Education and Promotion, School of Public Health, Iran University of Medical Sciences, Tehran, IR Iran; 4 Community Based Participatory Research Center, Iranian Institute for Reduction of High-Risk Behaviors, Tehran University of Medical Sciences, Tehran, IR Iran; 5 Research Center for Modeling in Health, Institute for Futures Studies in Health, Kerman University of Medical Sciences, Kerman, IR Iran; 6 Department of Epidemiology and Biostatistics, School of Public Health, Kerman University of Medical Sciences, Kerman, IR Iran

**Keywords:** Fatigue, Quality of Life, Multiple Sclerosis, Exercise Therapy

## Abstract

**Background:**

Multiple Sclerosis (MS) is a demyelinating disease of the nervous system which has numerous disabling effects on patients.

**Objectives:**

This study aimed at investigating the short- and long-term effects of a period of combination exercise therapy on walking distance, balance, fatigue and quality of life in multiple sclerosis patients referred to the physiotherapy clinic of Iran's Multiple Sclerosis Society in 2013.

**Patients and Methods:**

This study was a randomized controlled clinical trial on 59 patients divided into the intervention (n = 39) and control groups (n = 20). The intervention group received 10 weeks of combination therapy including aerobic, strengthening, balancing and stretching exercises. A week before, a week later and a year after the beginning of the exercises, both groups of patients received BBSS, six minute walking, Family Support Services (FSS), Expanded Disability Status Scale (EDSS) and quality of life tests. The scores of two groups were then compared using statistical tests such as repeated measures ANOVA test.

**Results:**

The results indicated significant changes in the intervention group in comparison to the control group in the second phase of the study comparing to the first one for all tests except EDSS (Mean difference scores of EDSS: -0.13), P-value = 0.60; FSS: -6.9, P-value = 0.02, Mental Quality of Life (QOL): 16.36, P-value = 0.001; Physical QOL: 12.17, P-value = 0.001, six minute walking: 137.2, P-value < 0.0001; and Berg: 3.34, P-value < 0.0001. These changes were not significant in the second phase of the study comparing to the third one; however, they were again significant in the third phase comparing to the first phase of the study (P < 0.05).

**Conclusions:**

Exercise has significant effect on improving symptoms of multiple sclerosis, and cessation of exercise may cause recurrence of symptoms in the intervention group with a slope similar to that of the control group. Therefore, continuous rather than short period exercises have valuable symptomatic and supportive relief effects in patients.

## 1. Background

Multiple sclerosis (MS) is an advanced neurologic disease which demyelinates the cells of central nervous system (1). Before 1970s, it was thought that any kind of physical movement and exercise may cause fatigue and new problems in the patient and therefore they must be avoided. However, it gradually appeared that physical exercises can be useful for the patients, reduce their fatigue, and increase their strength, perseverance, and life quality (2-4). The exercise used in these studies were a large range of exercises including strengthening exercises with weight lifting, perseverance and aerobics exercises on bicycle and treadmill, and various balancing and stretching exercises. For example, Ostert and Kesselring reported in 2001 from Switzerland, the improving of symptoms such as fatigue, physical activity level, and social activities in patients after four weeks of aerobics exercises (5). In a different report, however, Collett et al. from Oxford University declared that various exercises on a state bicycle didn't cause significant changes in their patients (6). In the first decade of the present century several reports were published regarding the positive effects of exercise on MS symptoms. White (2004) (7), Gutierrez (2005) in the United States (8), Taylor (2007) in Australia (9), De Souza (2009) in Spain (10), and Dalgas et al. (2010) in Denmark (11) maintained that increasing perseverance exercises could improve symptoms in multiple sclerosis patients. Moreover, Newman et al. in 2007 in Britain contended that exercise on treadmill improves walking distance and speed (12). Researchers gradually turned to combination exercise therapy and they could show these exercises have positive effects on patients' strength, perseverance, walking distance and quality of life. In Finland, for instance, Romberg et al. in 2004 reported that factors related to moving in multiple sclerosis patients improved after resistance and aerobics exercises, though these exercises did not have any effects on patients' quality of life (13). Australian researchers in Griffith University indicated in 2009 in a cross-sectional study that those patients with a higher activity level have less depression and fatigue issues. Also, Stroud et al. maintained that all aspects of quality of life in people who did regular exercises were significantly better from those of non-active people (14). Furthermore, in 2010, Cakit et al. in Turkey reported that exercises on state bicycles and balancing exercises improve factors such as fatigue and balance in multiple sclerosis patients and reduce the fear of falling in them (15). The good point of all these exercises was that they were easy for patients to perform and they could often finish the exercises successfully. Moreover, the results of most of these researches indicated the improvement of patients in performed items and tests. These exercises were usually performed from four to eight weeks (5, 7-13, 15) and only Romberg's lasted for six months (13). On the other hand, to measure the success of interventions, patients in most of these studies received tests immediately after they had completed the exercises and therefore the durability of the effects of these exercises were not measured. In other words, the long-term effect of these exercises on patients' personal lives were not investigated. There have been fairly few interventions investigating the long-term effects of exercises. McCullagh et al. in 2008 in Ireland, for instance, reported the long-term durability (six months) of the effects of exercises in reducing fatigue and increasing quality of life (16). Stuifbergen et al. in 2006 in the United States showed in a longitudinal qualitative study that there is a positive relationship between exercise and quality of life in multiple sclerosis patients. This study, which lasted for five years and during which 611 patients were regularly assessed, contended that patients with more physical exercise and activity were less vulnerable to the disabilities of the disease (17). Eventually, researchers such as Motl in 2008 (18), Snook in 2009 (19) and Asano et al. (20) in the same year, as well as Andreasen in 2013 (21) had a review on rehabilitation and exercise articles and interventions and their effects on MS, and declared that exercise therapy had more or less significant effects in reducing the problems caused by the disease. Although larger and better designed clinical trials are needed to prove this notion.

## 2. Objectives

The aim of this study was to investigate whether combination exercise therapy has long-term positive effects on patients and whether a period of short-term exercise can change patients' behavior for doing daily physical exercises. 

## 3. Patients and Methods

### 3.1. Study Population and Sampling

This study is a randomized clinical trial containing control and intervention groups and subjects were selected using simple randomization method. The participants consisted of 147 patients with multiple sclerosis which enrolled in this study based on convenience sampling method who were referred by neurologists to physiotherapy clinic of Iran's Multiple Sclerosis Society (located in Tehran, Iran) during a 14-months period from September 2012 to December 2013.

### 3.2. Sample Size

In this study, the total sample size was determined to be 42 for each intervention and control group and was calculated based on the prior studies considering 95% confidence level, 90% power, d = 1, and σ^2^ = 2. We used the below formula:

$$n=\frac{2{(Z_{\frac{1-\alpha }{2}}+Z_{1-\beta })}^{2}\times \sigma \times \sigma }{d^{2}}$$

### 3.3. Iran Multiple Sclerosis Society

MS society which is located in Tehran –the capital city of Iran, was launched in June 1999 and registered as a non-governmental charity in 2000. By now, it has about 18,000 members across the country. The first activity of the society was to establish a free of charge social and psychological consultation service for the patients. Then, this society extended its activities to preparation of informative brochures, following-up the treatment needs of patients, precise registration of patients’ information in aggregated databases, communication with the scientific centers and media around the world specially the Multiple Sclerosis International Federation (MSIF), holding various conferences, writing, translating and publishing books and related materials, collaboration with multiple research projects and launching the physiotherapy department. Furthermore, obtaining affordable MS drugs for patients through the governmental subsidies and minimizing treatment costs are considered substantial achievements of this NGO (22).

The participants were required to meet the following criteria: suffering from recurrent and improving type of MS, 18 to 50 years old, not having had any MS attack in the last three months and consuming various types of interferon for prevention of MS attacks (1). Also, these patients had to have EDSS scores of 0-4 (23), and higher scores excluded the patient from the research. Eighty four patients passed this first test. Then, using random number tables, these patients were divided into two groups: 42 entered the intervention group and the rest were assigned to the control group. Among the 42 patients in the control group, 30 patients accepted the invitation to take the test and thus as the control group, they conducted the initial experiments. According to the information obtained from patients in the intervention group and also the database of Iran's Multiple Sclerosis Society, patients in the control group matched those in the intervention group regarding their age, gender, disease type and intensity, and medications used. These were the patients who, due to the long distance or personal or occupational problems, were not definitely able to attend the center's routine or intervention sessions and it was certainly impossible to perform rehabilitation therapy for them. Their working hours, for instance, did not match the center's working hours, or the long distance between where they lived and the center’s location did not allow them to regularly attend this exercise period in the center. Their selection for inclusion in the control group was also ethically justified. These people had to avoid doing any planned physical or rehabilitation activity for 10 weeks. After the second phase of experiments, however, these people were also allowed to do exercises or different rehabilitation activities because it was not ethically feasible to deprive them from this type of treatment. In the intervention group, if patients experienced sudden attacks during the study period or did not attend the required number of exercise sessions, they were removed from the study.

### 3.4. Data Gathering Instruments

The experiment in this research was conducted in three phases: once a week before the exercise, the second time a week after ten weeks of exercise, and the third phase was done after all exercise sessions. These tests included measurement of disability in MS patients (23), level of balance (Persian version of Berg balance test) (24), walking distance (6-min walking test) (25), fatigue intensity test (26, 27), and quality of life test specific for MS patients (28). Also, in the third test the number of possible attacks in the last nine months was reported by patients themselves. Patients' scores were reported by a neurologist. Six-min walking test was conducted in a gymnasium outside the place where exercises were done. Using computerized versions, other tests were done by a skilled trainer different from main trainers and researchers in the exercise area. The results were automatically generated by a computer and without any interventions by the researchers.

### 3.5. Intervention

The intervention in this research included 10 weeks of combination exercises including stretching and aerobics exercises, strengthening exercises with spring, and balancing exercises with tilt board and cerebral palsy ball. Three exercise sessions per week with a total number of 30 sessions were considered for the patients. At the beginning of every session, patients, with the guidance of a therapist, performed the stretching exercises including movements for spine and neck and upper and lower limbs. These exercises took seven to ten minutes. Then, in every exercise session, patients used a combination of state bicycle and treadmill to do aerobics exercises. These sessions started with 20 minutes exercise for the first session and then were increased in time. Depending on the patients' fatigue, these exercises took up to 40 minutes in every session. The time of aerobics exercises was divided equally between bicycle and treadmill. The difficulty level in every session started from a low point and gradually reached to the climax and once again decreased and returned to the starting point. Aerobics exercises in the first session included 10 minutes exercise on state bicycle with 40 percent of the maximum heart beat, 10 minutes walking on treadmill with 40 percent of the maximum heart beat, and 10 minutes non-active rest between them. In the last session, these activities took 20 minutes with 70 percent of maximum heart beat. During aerobics exercises, patients were repeatedly recommended to rest before they feel exhausted. In every treatment session, patients did strengthening exercises with a spring for strengthening their quadriceps, gluteal, and cuff muscles. Exercise regimen for these muscles started many cycles of low intensity exercise and took nearly 10-15 minutes. In every session, patients did various balancing exercises with circular and rectangular tilt boards and also cerebral palsy ball. These exercises took 10 minutes at the beginning and gradually increased to 20 minutes. These exercises included standing with one foot and two feet on rectangular tilt board at the beginning and after some sessions patients exercised with the circular type device. Furthermore, sitting on cerebral palsy balls and doing exercises on them with one foot or two feets by patients was considered as a balancing exercise. Thus, every session started with one active hour and gradually, depending on patients' endurance, increased to nearly 90 minutes. Patients were allowed to take enough rest between exercises to refresh themselves and overcome their fatigue. Whenever possible they were offered proper fruit juice, biscuits, and dates. It must also be noted that patients received sufficient explanation about the methods, principles, and benefits of exercises for MS patients and were encouraged to do exercises on a regular and long-term basis (29).

### 3.6. Ethical Considerations

Ethical issues (including plagiarism, informed consent, research misconduct, data fabrication and/or falsification, double publication and/or submission, redundancy, etc.) have been completely observed by the authors. The Ethics Committee of Kerman University of Medical Sciences approved the study protocol with Code No: K/92/398. For ethical reasons, at the end of the study the control group received also combination exercises. Informed consent (oral and written) of all participants was obtained and regulations of the Declaration of Helsinki was followed throughout the study.

### 3.7. Analysis

The normality of data was tested and confirmed by Kolmogorov-Smirnov test. The analysis was done using descriptive statistical methods such as average standard deviation and analytical statistics such as repeated measurement to investigate the effect of repetitive variables in the control and intervention groups and in three phases (phase one, phase two, and phase three). Also the simultaneous effect of group and time variables (interaction effect) were analyzed. In this research, average scores of disability, walking distance, fatigue intensity, balance level, and physical and mental quality of life were compared between intervention and control groups in three time points throughout the study (a week before, a week after, and a year after the intervention). The effect of other factors such as gender (male-female), age, and number of attacks as confounding factors were entered into the model. The analysis was done using SPSS 20 and significance level was considered to be P-values < 0.05.

## 4. Results

Thirty nine (15 men and 24 women) out of 42 patients in the intervention group managed to finish the exercises and entered the second phase of experiments. One patient left the exercises unfinished due to lack of enough time. One patient stopped performing exercises because of muscular pain in cuff muscles and one other patient was removed from the study due to a new attack and non-responding symptoms. In the third phase, four patients were unavailable because they went on trips or changed their addresses. Eventually, 35 patients finished this test completely. Also, 22 (7 men and 15 women) out of 30 patients in the control group in the initial phase could complete the second-phase tests and eight were removed from the study. Five patients were removed because they did perform exercises or began rehabilitation, one because of a new attack or severity of symptoms, and two because of personal problems. Moreover, two patients could not be invited to the third phase of experiments because they had changed their addresses. Finally, the scores of 20 patients were included in the final analysis. Table 1 shows the characteristics of patients in the intervention and control groups.

**Table 1.** The Distribution of Demographic Variables in the Two Study Groups a

Groups	Male	Female	Total	Age	Number of Attacks (After Intervention)
**Intervention Group**	15	24	39	33.05 ± 7.68	18
**Control Group**	7	15	22	32.05 ± 6.35	12
**Total**	22	39	61	-	-

^a^ Data are presented as mean ± SD.

### 4.1. Tests Results

#### 4.1.1. EDSS Test

**Figure 1.**
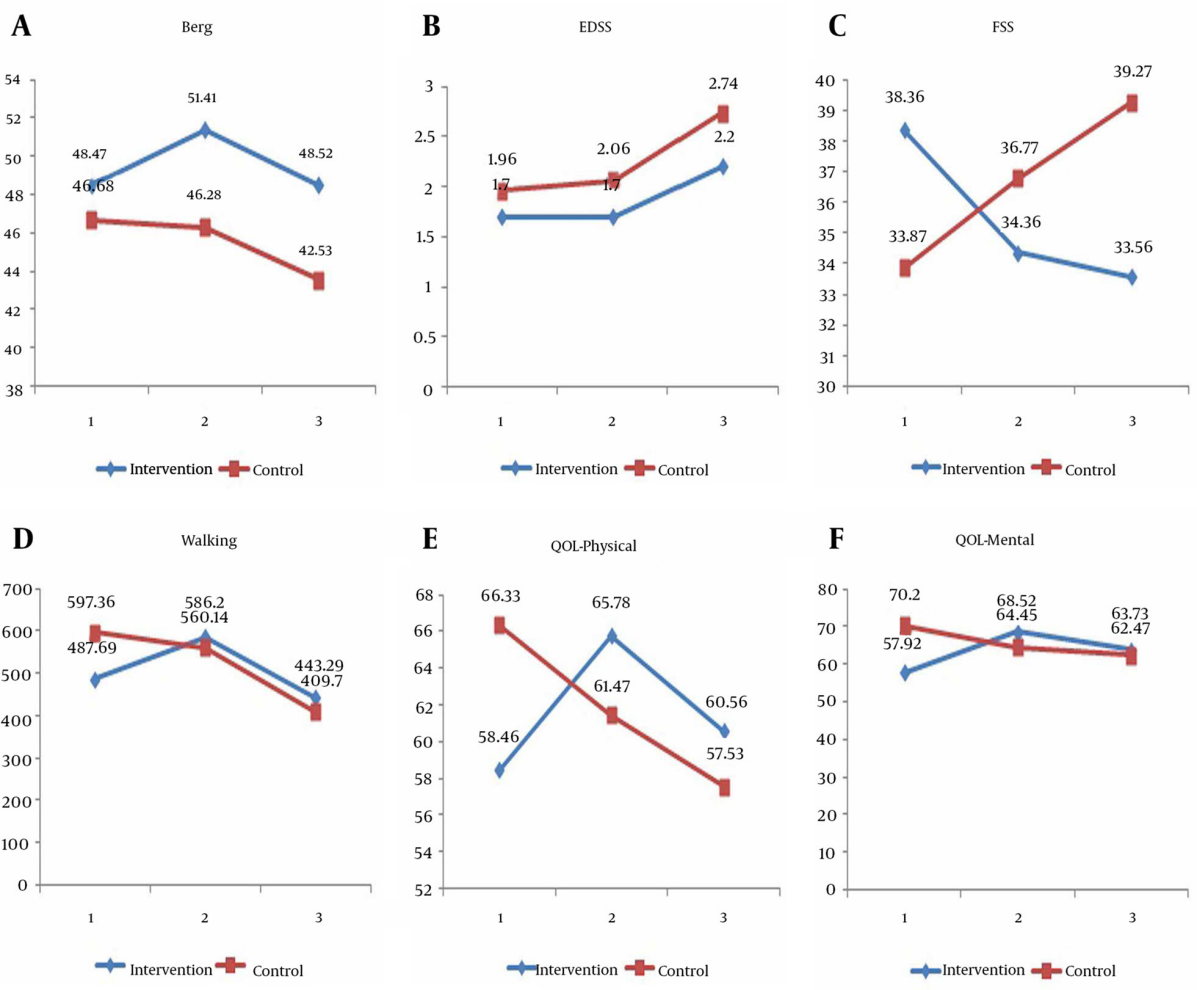
Comparing the Mean EDSS, Berg, six Minutes Walking, FSS, Physical and Mental Quality of Life Test Scores in the First, Second and Third Time Points in the Intervention and Control Groups

In the EDSS test, there were no significant changes in the first and second periods in the two groups as compared within the group, but in the third period both groups showed a significant increase in the scores of this test. This increase was about half a point for the intervention group (P = 0.001) and 0.72 for the control group (P = 0.001) (Figure 1). Also, results of the analysis concerning the comparison of groups' average changes in various time points were not significant (Table 2) .

#### 4.1.2. Fatigue Test

In the fatigue test, a significant decrease in the scores was observed in the second (P = 0.02) and third time points (P = 0.04) compared to the baseline (day-zero). In the control group, however, changes of fatigue scores in comparing the three time periods of the test were not significant (Figure 1). The significance of changes in two groups as compared with each other, was also statistically tested. According to this comparison, the degree of changes in the second part of time as compared to the first part was significant in the intervention group when compared with the control one (P = 0.02). Also the values in the third time point were significantly different from the first time point (P = 0.004), but results and changes in second and third time points were not significantly different (P = 0.30) (Table 2).

**Table 2.** The Mean Difference Scores of EDSS, FSS, Mental QOL, Physical QOL, Six Minute Walking and BBS in the two Study Groups at Three Time Points ^a^

Variable	Change 1 ^b^		Change 2 ^c^		Change 3 ^d^	
	Mean Difference ± SD ^e^	P Value	Mean Difference ± SD	P Value	Mean Difference ± SD	P Value
**EDSS**	-0.13 ± 0.23	0.60	-0.15 ± 0.21	0.50	-0.28 ± 0.29	0.35
**FSS**	-6.9 ± 2.82	0.02	-3.3 ± 3.18	0.30	-10.2 ± 3.42	0.004
**Mental QOL**	16.36 ± 4.46	0.001	2.82 ± 4.85	0.56	13.54 ± 5.37	0.02
**Physical QOL**	12.17 ± 3.62	0.001	-1.27 ± 3.61	0.73	10.90 ± 4.55	0.02
**6 minute Walking**	137.2 ± 24.54	<0.0001	47.07 ± 45.34	0.30	184.3 ± 51.1	0.001
**Berg**	3.34 ± 0.87	<0.0001	-0.14 ± 1.32	0.92	3.21 ± 1.44	0.03

^a^ Abbreviations: EDSS; Expanded Disability Status Scale, FSS; Family Support Services, QOL; Quality of Life.

^b^ Mean change first test Mean change second test = change-1.

^c^ Mean change second test Mean change third test = change-2.

^d^ Mean change first test Mean change third test = change-3.

^e^ Mean intervention group Mean control group = Mean difference.

#### 4.1.3. Quality of Life Tests

Concerning the mental quality of life, significant changes were observed in the intervention group in the time period as compared with the first and third time periods (P < 0.05). In the control group, however, results of all time periods were not statistically different from each other (Figure 1). The changes in the two groups were also statistically compared. According to this comparison, the changes in the second time period was compared to the first time period which was significantly different in the intervention group as compared with control group (P = 0.001). The levels for the third time period was also significantly different from the first time period (P = 0.02), but changes in the second part as compared with the third part were not statistically significant (P = 0.56) (Table 2). Concerning the physical quality of life, significant changes were observed in the intervention group in the second time period as compared with the first time period (P = 0.001). After nine months, however, the values returned to their initial level and thus there was no statistically significant difference between first and third parts. In the control group, values decreased significantly when comparing the third time period with the first time period (P = 0.02) (Figure 1). Comparison of changes between the two groups also indicated that the values of second time period as compared to the first time period and the third time period as compared to the first time period had significant changes (P < 0.05) (Table 2). 

#### 4.1.4. Six Minutes Walking Test

The average scores in the two study groups in three time periods showed that the differences of average scores of walking distance in the first and second time periods and also in the second and third time periods were statistically significant in intervention group (P < 0.0001). The changes were also statistically significant in control group in the first and third time periods and in the second and third time periods as well (P < 0.0001) (Figure 1). The comparison of changes in the two groups in different time periods indicated that changes were significant when comparing the first part with the second time periods (P < 0.0001) and also comparing the first and the third time periods (P = 0.001) (Table 2).

#### 4.1.5. Berg Test

The results of Berg test showed an increasing and then decreasing pattern in the intervention group. In this group, significant positive changes were observed in the second phase as compared to the first and third phases. The mean score of Berg test increased from 49.02 to 51.7 which were significantly different. This score decreased to 48.63 in the third phase which was again statistically significant. In the control group, however, these changes were observed in a general decreasing pattern. Average scores were not significantly different when comparing the first and second test phases, but in the third phase, it had a decrease of 3.1 points as compared to the second phase which was statistically significant (Figure 1). The significance of changes in the two groups was also statistically tested. According to this comparison, the amount of changes in the second time period as compared to the first time period was significant in the intervention group when compared with the control group (P < 0.0001). Changes in the third time period were also significantly different from the first time period (P = 0.03), but the changes in second and third time periods were not significantly different (P = 0.92) (Table 2).

## 5. Discussion

It seems that there was a significant improvement in the intervention group in the second part of the test which was reversed in the third part when exercises were stopped and almost most test results returned to their base level. When compared with the control group which had a tangible decrease at least in the third part as compared with the first part, it can be concluded that rehabilitation and exercise therapy can help patients by decreasing the disease related complications at least in short term. These results were in line with most recent studies, though the effects of exercise cessation were not studied by any of these researchers. Our findings were in accordance with the studies of Dalgas in 2012 (30), McCullagh in 2008 (16), Stuifbergen in 2006 (17) and Romberg in 2005 (13). In our study, results of EDSS test in two groups as compared with each other at all-time points were not significant. This finding was in line with many previous studies including that of Pilutti in 2011 (31), Petajan in 1996 (32) and Rodgers in 1999 (33) which had used perseverance exercises and that of Dalgas in 2009 (34), Fimland in 2010 (35) and White in 2004 (7) which had used strengthening exercises, or that of Romberg in 2004 (13) and Bjarnadottir in 2007 (36) which had used combination exercises. Only one study by Golzari et al. in 2010 (37) which was performed using combination exercises reported a significant improvement in EDSS scores. Perhaps the most and clearest amount of changes in this research was regarding the Berg balancing test. Changes in this regard were highly significant and patients experienced an improvement in their balance. Results of our study matches those of other studies including that of D. Cattaneo in 2007 (38), Masoudinejad et al. in 2012 (39), Learmonth et al. in 2012 (40), Sosnoff et al. in 2013 (41) and Tarakci et al. in 2013 (42) which showed an improvement in balance and also a decrease in the risk of falling after the exercises. De Bolt, however, in 2004 showed that strengthening exercises have no effects on the balance level (43). This discrepancy between the results can be due to the differences in the type of exercises undertaken and also different tools for measuring the balance level in patients. The difference in the scores of two groups which reached nearly four points is noteworthy. This was in line with the study of Donoghue et al. in 2009 (44) which investigated what degree of changes in Berg test would correspond to real changes in patients. Regarding the fatigue test, patients in intervention group improved significantly as compared with control group. These results can be interpreted from two aspects: first, fatigue is a consequence of the disease process which will not be changed significantly by exercise. Studies of Pilutti in 2013 (31) and Andreasen in 2013 (21) support this notion. Also fatigue can be regarded in the context of patients’ general health status which can be enhanced following the improvement in patients’ general condition. Study of Dalgas et al. in 2012 support this notion (30). The changes in the six minute walking followed the general trend of this study and showed an increase in the second phase and a decrease in the third phase. These results matched those of Tarakci et al. in 2013 (42) but did not match those of Learmonth in 2012 (40). It should be mentioned that in these rather similar studies, mostly the speed of taking steps was measured and not the walking distance. Indeed, the analysis of Snook et al. in 2008 (19), confirmed the effect of exercise therapy on improving the walking distance though the changes were rather minor. In measuring the patient’ quality of lives, the results showed that there have been significant changes in the intervention group as compared with the control group in the second phase of the study compared with the first phase and the third phase as compared with the first in both physical and mental health aspects of quality of life. These results matched those of Mostert in 2001 (5) and Motl in 2008 (18), but Romberg in 2005 (13) reported in a longitudinal study that exercise was not effective in enhancing the quality of life in MS patients. The difference in tools which measure the quality of life in MS patients such as MSQOL-54 and other general tools can cause such difference (30). Type and duration of exercises are among other differences which should be mentioned (30).

Our research showed exercise therapy and sports are vitally valuable for patients and exercise causes noticeable improvement in them. This research also showed that, over time, there were significant differences in the quality of life between those patients who exercised and those who were deprived from rehabilitation and exercise therapy for some reasons. In the intervention group after nine months, the test scores returned to the base level. Comparing this with the control group which had a significant decrease from the base level, it can be concluded that rehabilitation can be crucially important in keeping MS patients capable of doing their daily activities. As the most important result of this research, it is necessary to mention that exercise cessation and the lack of follow-up causes the obvious return of the symptoms and disabilities and this emphasizes the necessity of doing exercises regularly and continuously. More longitudinal studies are required to design a proper, balanced and effective exercise regimen for the patients and help them fight the complications of their disease.

## References

[1] Umphred DA, Lazaro RT, Roller M, Burton G (2013). Neurological rehabilitation..

[2] Rietberg MB, Brooks D, Uitdehaag BM, Kwakkel G (2005). Exercise therapy for multiple sclerosis.. Cochrane Database Syst Rev..

[3] Petajan JH, White AT (1999). Recommendations for physical activity in patients with multiple sclerosis.. Sports Med..

[4] Solari A, Filippini G, Gasco P, Colla L, Salmaggi A, La Mantia L (1999). Physical rehabilitation has a positive effect on disability in multiple sclerosis patients.. Neurology..

[5] Mostert S, Kesselring J (2002). Effects of a short-term exercise training program on aerobic fitness, fatigue, health perception and activity level of subjects with multiple sclerosis.. Mult Scler..

[6] Collett J, Dawes H, Meaney A, Sackley C, Barker K, Wade D (2011). Exercise for multiple sclerosis: a single-blind randomized trial comparing three exercise intensities.. Mult Scler..

[7] White LJ, McCoy SC, Castellano V, Gutierrez G, Stevens JE, Walter GA (2004). Resistance training improves strength and functional capacity in persons with multiple sclerosis.. Mult Scler..

[8] Gutierrez GM, Chow JW, Tillman MD, McCoy SC, Castellano V, White LJ (2005). Resistance training improves gait kinematics in persons with multiple sclerosis.. Arch Phys Med Rehabil..

[9] Taylor NF, Dodd KJ, Prasad D, Denisenko S (2006). Progressive resistance exercise for people with multiple sclerosis.. Disabil Rehabil..

[10] de Souza-Teixeira F, Costilla S, Ayan C, Garcia-Lopez D, Gonzalez-Gallego J, de Paz JA (2009). Effects of resistance training in multiple sclerosis.. Int J Sports Med..

[11] Dalgas U, Stenager E, Jakobsen J, Petersen T, Hansen HJ, Knudsen C (2010). Fatigue, mood and quality of life improve in MS patients after progressive resistance training.. Mult Scler..

[12] Newman MA, Dawes H, van den Berg M, Wade DT, Burridge J, Izadi H (2007). Can aerobic treadmill training reduce the effort of walking and fatigue in people with multiple sclerosis: a pilot study.. Mult Scler..

[13] Romberg A, Virtanen A, Ruutiainen J (2005). Long-term exercise improves functional impairment but not quality of life in multiple sclerosis.. J Neurol..

[14] Stroud NM, Minahan CL (2009). The impact of regular physical activity on fatigue, depression and quality of life in persons with multiple sclerosis.. Health Qual Life Outcomes..

[15] Cakt BD, Nacir B, Genc H, Saracoglu M, Karagoz A, Erdem HR (2010). Cycling progressive resistance training for people with multiple sclerosis: a randomized controlled study.. Am J Phys Med Rehabil..

[16] McCullagh R, Fitzgerald AP, Murphy RP, Cooke G (2008). Long-term benefits of exercising on quality of life and fatigue in multiple sclerosis patients with mild disability: a pilot study.. Clin Rehabil..

[17] Stuifbergen AK, Blozis SA, Harrison TC, Becker HA (2006). Exercise, functional limitations, and quality of life: A longitudinal study of persons with multiple sclerosis.. Arch Phys Med Rehabil..

[18] Motl RW, Gosney JL (2008). Effect of exercise training on quality of life in multiple sclerosis: a meta-analysis.. Mult Scler..

[19] Snook EM, Motl RW (2009). Effect of exercise training on walking mobility in multiple sclerosis: a meta-analysis.. Neurorehabil Neural Repair..

[20] Asano M, Dawes DJ, Arafah A, Moriello C, Mayo NE (2009). What does a structured review of the effectiveness of exercise interventions for persons with multiple sclerosis tell us about the challenges of designing trials?. Mult Scler..

[21] Andreasen AK, Stenager E, Dalgas U (2011). The effect of exercise therapy on fatigue in multiple sclerosis.. Mult Scler..

[22] Sahraian MA, Khorramnia S, Ebrahim MM, Moinfar Z, Lotfi J, Pakdaman H (2010). Multiple sclerosis in Iran: a demographic study of 8,000 patients and changes over time.. Eur Neurol..

[23] Kurtzke JF (1983). Rating neurologic impairment in multiple sclerosis: an expanded disability status scale (EDSS).. Neurology..

[24] Azad A, Taghizadeh G, Khaneghini A (2011). Assessments of the reliability of the Iranian version of the Berg Balance Scale in patients with multiple sclerosis.. Acta Neurol Taiwan..

[25] Savci S, Inal-Ince D, Arikan H, Guclu-Gunduz A, Cetisli-Korkmaz N, Armutlu K (2005). Six-minute walk distance as a measure of functional exercise capacity in multiple sclerosis.. Disabil Rehabil..

[26] Krupp LB, LaRocca NG, Muir-Nash J, Steinberg AD (1989). The fatigue severity scale. Application to patients with multiple sclerosis and systemic lupus erythematosus.. Arch Neurol..

[27] Chipchase SY, Lincoln NB, Radford KA (2003). Measuring fatigue in people with multiple sclerosis.. Disabil Rehabil..

[28] Ghaem H, Borhani Haghighi A, Jafari P, Nikseresht AR (2007). Validity and reliability of the Persian version of the multiple sclerosis quality of life questionnaire.. Neurol India..

[29] Sangelaji B, Hatamizadeh N, Rashvand F, Kazemnejad A (2011). Study about the effects of rehabilitation on quality of life in multiple sclerosis patients. J Nurs Midwife.

[30] Dalgas U, Stenager E (2012). Exercise and disease progression in multiple sclerosis: can exercise slow down the progression of multiple sclerosis?. Ther Adv Neurol Disord..

[31] Pilutti LA, Lelli DA, Paulseth JE, Crome M, Jiang S, Rathbone MP (2011). Effects of 12 weeks of supported treadmill training on functional ability and quality of life in progressive multiple sclerosis: a pilot study.. Arch Phys Med Rehabil..

[32] Petajan JH, Gappmaier E, White AT, Spencer MK, Mino L, Hicks RW (1996). Impact of aerobic training on fitness and quality of life in multiple sclerosis.. Ann Neurol..

[33] Rodgers MM, Mulcare JA, King DL, Mathews T, Gupta SC, Glaser RM (1999). Gait characteristics of individuals with multiple sclerosis before and after a 6-month aerobic training program.. J Rehabil Res Dev..

[34] Dalgas U, Stenager E, Jakobsen J, Petersen T, Hansen HJ, Knudsen C (2009). Resistance training improves muscle strength and functional capacity in multiple sclerosis.. Neurology..

[35] Fimland MS, Helgerud J, Gruber M, Leivseth G, Hoff J (2010). Enhanced neural drive after maximal strength training in multiple sclerosis patients.. Eur J Appl Physiol..

[36] Bjarnadottir OH, Konradsdottir AD, Reynisdottir K, Olafsson E (2007). Multiple sclerosis and brief moderate exercise. A randomised study.. Mult Scler..

[37] Golzari Z, Shabkhiz F, Soudi S, Kordi MR, Hashemi SM (2010). Combined exercise training reduces IFN-gamma and IL-17 levels in the plasma and the supernatant of peripheral blood mononuclear cells in women with multiple sclerosis.. Int Immunopharmacol..

[38] Cattaneo D, Jonsdottir J, Zocchi M, Regola A (2007). Effects of balance exercises on people with multiple sclerosis: a pilot study.. Clin Rehabil..

[39] Masuodi Nezhad M, Shivani H, Hossini F (2012). Effects of selected combined training on balance and functional capacity in women with multiple sclerosis.. WASJ..

[40] Learmonth YC, Paul L, Miller L, Mattison P, McFadyen AK (2012). The effects of a 12-week leisure centre-based, group exercise intervention for people moderately affected with multiple sclerosis: a randomized controlled pilot study.. Clin Rehabil..

[41] Sosnoff JJ, Finlayson M, McAuley E, Morrison S, Motl RW (2014). Home-based exercise program and fall-risk reduction in older adults with multiple sclerosis: phase 1 randomized controlled trial.. Clin Rehabil..

[42] Tarakci E, Yeldan I, Huseyinsinoglu BE, Zenginler Y, Eraksoy M (2013). Group exercise training for balance, functional status, spasticity, fatigue and quality of life in multiple sclerosis: a randomized controlled trial.. Clin Rehabil..

[43] DeBolt LS, McCubbin JA (2004). The effects of home-based resistance exercise on balance, power, and mobility in adults with multiple sclerosis.. Arch Phys Med Rehabil..

[44] Donoghue D, Physiotherapy R, Older People G, Stokes EK (2009). How much change is true change? The minimum detectable change of the Berg Balance Scale in elderly people.. J Rehabil Med..

